# Long Term Outcome of 112 Pediatric Patients With Ureteroplevic Junction Obstruction Treated by Endourologic Retrograde Balloon Dilatation

**DOI:** 10.3389/fped.2022.863625

**Published:** 2022-04-25

**Authors:** Javier Ordóñez, Rubén Ortiz, Alberto Parente, Laura Burgos, Beatriz Fernández-Bautista, Laura Pérez-Egido, José María Angulo

**Affiliations:** ^1^Pediatric Urology, Hospital General Universitario Gregorio Marañón, Madrid, Spain; ^2^Reina Sofía University Hospital, Córdoba, Spain

**Keywords:** endourology, ureteropelvic junction obstruction, high-pressure balloon dilatation, pediatric urology, minimally invasive approach

## Abstract

**Purpose:**

To analyze the effectiveness, complications and long-term outcome of the patients with ureteropelvic junction obstruction (UPJO) treated by endoscopic retrograde balloon dilatation (ERBD) in the largest series reported.

**Materials and Methods:**

Between years 2004 and 2018, 112 patients with primary unilateral UPJO were treated by ERBD. Endoscopic treatment consisted on a retrograde balloon dilatation of the ureteropelvic junction (UPJ), through cystoscopy and under fluoroscopic guidance, using high-pressure balloon catheters. In case of persistence in the balloon notch, a Cutting Balloon™ catheter was used. Double-J stent was placed after dilatation.

**Results:**

Mean age at surgery was 13.1 ± 21.3 months, 92 cases being younger than 18 months. Mean operative time was 24.4 ± 10.3 min; hospital stay was 1 day in 82% of patients. No intraoperative complications occurred. UPJ was calibrated at time of stent removal with cystoscopy 39.1 ± 13.7 days after dilatation. ERBD was not possible in 11 cases. An additional procedure was needed in 24 cases: second ERBD (*n* = 11, seven during the stent withdrawal), a third dilatation (*n* = 3) due to persistent hydronephrosis, and percutaneous endopyelotomy (*n* = 3) or open pyeloplasty (*n* = 7) in cases of technical failure. Significant improvement in postoperative ultrasound measures were observed (*p* < 0.05, T-test). Long-term success rate was 76.8% after one dilatation, and 86.6% in those who required up to 2 dilatations. Mean follow-up was 66.7 ± 37.5 months.

**Conclusions:**

ERBD is a feasible and safe option for the minimally invasive treatment of UPJ obstruction in infants. Long-term outcome is acceptable with a very low complication rate.

## Introduction

The treatment of the ureteropelvic junction obstruction (UPJO) in children is changing in recent years, with a greater tendency to perform minimally invasive approaches. Open pyeloplasty has demonstrated a success rate of over 94% (1, 2). Recent publications report an increasing effectiveness of the robot-assisted (3), laparoscopic (4) and endourologic approaches (5, 6). The

principal advantages of these techniques (reduction of postoperative pain, length of hospital stay, better cosmesis, etc.), together with their effectiveness and safety, are making them considered as the first therapeutic option in many cases (7, 8).

Endourologic balloon dilatation for the treatment of UPJO, widely used in the adult population, was first described in 1982 (9–11). The experience and outcomes in children are more limited. Pediatric sized instruments and technical improvement in the last years made it a safe and feasible approach for small children. Thus, the endourologic approach has been successfully used in the treatment of other obstructive urologic conditions [such as the primary obstructive megaureter (12, 13)], and in secondary UPJO (14–16). But the role of the endourologic treatment for the primary UPJO has been largely questioned, with discrepancy in the success rates and outcomes published (5, 6, 17).

In the present study, we report our experience in the management of primary UPJO treated by endourologic retrograde balloon dilatation (ERBD), which is established as first line of treatment in our institution (5). Furthermore, the peripheral cutting balloon microsurgical dilatation device (18) (Cutting Balloon™, CB) has been used in those patients that presented an incomplete resolution of the narrowing during the high-pressure balloon (HPB) dilatation. The aim of the study is to analyze the effectiveness, complications, and long-term outcomes of ERBD of primary UPJO in the largest pediatric series reported.

## Materials and Methods

After the institutional review board approval, we retrospectively analyzed all pediatric patients with unilateral primary UPJO treated by ERBD between July 2004 and September 2018 in our institution. Exclusion criteria were bilateral UPJO requiring intervention, associated urological anomalies [vesicoureteral reflux, extrinsic UPJO (as the presence of a polar vessel), obstructive primary megaureter, ureterocele, etc.] and a postoperative follow-up period lower than 18 months.

Hydronephrosis grade was defined according to the guidelines of the Society of Fetal Urology Classification and Urinary Tract Dilatation Grading System (19, 20). Ultrasound scan (at second day of life in cases of prenatal diagnosis, 1 month of life and every 3 months, under conservative surveillance with low-dose antibiotic prophylaxis) was used to measure the anteroposterior pelvis diameter, calyces and renal parenchyma thickness. Preoperative ultrasound showed grade IV hydronephrosis in all patients. Mercaptoacetyltriglycine (MAG-3) renal scans with furosemide washout revealed obstructive pattern in all cases, with a washout halftime T1/2 > 50 min (21). In all cases, a micturating cystourethrogram was previously performed to rule out the presence of vesicoureteral reflux (exclusion criteria). During those years, all patients with primary UPJO were treated with ERBD.

Clinical data, ultrasonography images, scintigraphy scans, and outcome were preoperatively and post-operatively analyzed. Intraoperative and perioperative complications were assessed according Clavien-Dindo classification. Statistical analysis was performed using IMB^©^ SPSS^©^ Statistics Version 25. Analysis of continuous variables was performed using the *t*-student test.

### Technique Description

Under general anesthesia, with appropriate antibiotic prophylaxis and patient in lithotomy position, a cystoscopy is performed using a 9.5 Fr cystoscope with a 5 Fr instrumentation channel. A 4 Fr catheter is placed in the affected ureter in order to perform a retrograde pyelography. A hydrophilic guidewire (0.014” Choice PT™, J-tip, Boston Scientific; or 0.018” Radiofocus^®^ Terumo) is negotiated up to the renal pelvis. In case of difficulties, a 0.035” hydrophilic guidewire is used (it travels easier in the retrograde direction inside the ureteral lumen). Then, a 3 Fr high-pressure balloon catheter is inserted over the guidewire and located in the UPJ, under fluoroscopic guidance. The balloons used were semi-compliant dilatation catheters with nominal diameter of 5–7 mm according to the patient's weight (5 mm in patients <6 kg, 6 mm in 6–10 kg, and 7 mm in >10 kg) and 2 cm length (RX Muso™, *Terumo*). When the balloon is located at the UPJ, it is filled with radiologic contrast to its nominal pressure (14–16 atm) under fluoroscopic control until the balloon notch or hourglass image disappears. After successful dilatation procedure, a double-J ureteral stent is placed (3 Fr, 8–12 cm long; *Sof-Flex Multi-Length Ureteral Stents; CookMedical Europe*™) between renal pelvis and bladder. The transurethral bladder catheter is removed 16–18 h after surgery, and oral antibiotic prophylaxis is administered until the double-J catheter is removed.

The Cutting Balloon™ (Boston Scientific, Natick, MA, USA) catheter is reserved for those cases when the HPB notch or hourglass image does not completely disappear after 20 s at 16–18 atm. In those cases, a 3-, 4-, or 5-mm diameter CB and 2 cm length is inflated at the level of the ureteropelvic junction to up to 12 atm. Dilatation is then completed using a HPB as described before, and double-J ureteral stents is always placed. The antibiotic prophylaxis and urethral drainage protocol does not differ with respect to patients in whom HPB is used.

Double J stents are removed 4–6 weeks after the dilatation procedure and the UPJ is assessed (calibration) in day-hospital regimen. The calibration of the UPJ consist of the inflation of a balloon at low pressure (6–8 atm) in the UPJ to check the absence of residual stenosis. If a residual stenosis is found in the fluoroscopy, a new dilatation is then performed using an HPB. In these cases, a double-J stent is placed with a 4/0 Prolene suture attached to its distal tip and exteriorized (transurethral), being removed 1 week later in the outpatient clinic (pulling out the suture). Similar to the initial intervention, if the residual stenosis still persists after an HPB, a CB dilatation is then performed, and the double-J stent is removed in the daycare center 4 weeks later.

### Postoperative Control

**Figure 1:**
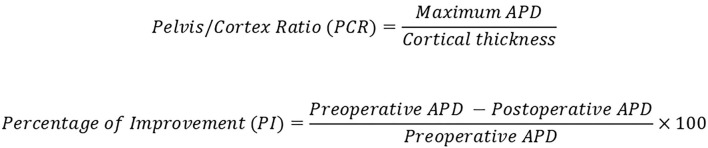
Pelvis/cortex ratio and percentage of improvement (PI) in the renal pelvis APD.

Follow-up consists in regular clinical review and renal ultrasonography at 3, 6, and 12 postoperative months, and then each 6 months until clinical and radiologic stabilization is observed. Antero-posterior renal pelvis diameter (APD, maximum pelvis diameter in a coronal view), pelvis/cortex ratio (PCR, Figure 1) and percentage of improvement (PI, Figure 1) of the APD are the main parameters used to predict the outcome. Postoperative MAG-3 diuretic renogram is performed only when a poor outcome is predicted at the six postoperative months attending to the ultrasonography parameters (when APD > 18.5 mm, PI <35%, or PCR > 3.5). In case of UPJO recurrence, several minimally invasive options are posed: a new ERBD, CB dilatation or percutaneous anterograde endopyelotomy. In case of not being resolutive, an open dismembered pyeloplasty is performed and considered as a failure of the endourologic strategy.

## Results

Between July 2004 and September 2018, 112 patients with unilateral primary UPJO were treated with ERBD and met the inclusion criteria. Prenatal diagnosis of hydronephrosis was presented in 98 (87.5%) of them. Indications for surgery are shown in Table 1. Demographics, preoperative ultrasound findings and mean postoperative follow-up time are shown in Table 2. Mean follow-up time was 66.7 ± 37.5 months. A crossing lower pole vessel (exclusion criteria) was detected postoperatively in six cases (bad evolution after an initial ERBD), and in four cases the diagnosis was made initially due to a high degree of clinical and radiological suspicion.

**Table 1:** Indications for surgery.

**Indications for surgery**	***n***
Grade IV hydronephrosis and obstructive diuretic drainage curve	All cases (*n* = 112)
Differential renal function <40%	*n* = 24
Increased hydronephrosis worsening with renal parenchyma thinning	*n* = 76
Loss of renal function >10% during expectative surveillance	*n* = 2
Recurrent febrile urinary tract infections	*n* = 10

**Table 2:** Demographics, age at surgery, preoperative ultrasound findings and follow-up time of patients who met the inclusion criteria.

**Sex**	**Male = 83 (74.1%)**
	**Female = 29 (25.9%)**
**Mean age at surgery (mean, SD)**	**13.1 ± 21.3 months (*n* = 92 under 18 months of age)**
**Preoperative anteroposterior pelvis diameter** **(mean, SD)**	**25.3 ± 9.8 mm**
**Preoperative parenchymal thickness (mean, SD)**	**4.2 ± 1.6 mm**
**Follow-up time (mean, SD)**	**66.7 ± 37.5 months**

Dilatation was successfully performed in 101 cases (90.2%). In 11 of these cases, the use of CB was needed in order to complete the dilatation. The diameters of HPB catheters used were 5 mm (*n* = 38), 6 mm (*n* = 51) and 7 mm (*n* = 12); diameters of CB were 3 mm (*n* = 3), 4 mm (*n* = 7), and 5 mm (*n* = 1). Mean surgical time was 24.4 ± 10.3 min, and there were no intraoperative complications. In 11 patients (9.8%), the retrograde HPB dilatation was not successful; causes of the failure and outcomes of these patients are shown in Table 3. Hospital stay was 24 h in 92 patients (82.1%), 48 h in 15 (13.4%) and more than 48 h in 5 (4.5%) due to preoperative urinary tract infection requiring intravenous antibiotics (*n* = 3) and to pain and vomiting (*n* = 2). In all patients, the analgesic medication consisted exclusively in non-steroidal anti-inflammatory drugs (no need of opioids). No oral analgesics were needed after discharge.

**Table 3:** Causes of failure in the first intervention and calibration, and outcome of these patients.

**Intervention**	**Incidence**	**Number of cases**	**Outcome (number of cases)**
First intervention	Persistence of hourglass image	9	Second HPBD (1) More than 2 dilatations (2) Second dilatation with Cutting Balloon (4) Open pyeloplasty (2)
	Failure at double-J insertion	1	Open pyeloplasty (1)
	Failure to place the guidewire in the renal pelvis	1	Pyeloplasty (1)
Calibration	Failure to place the guidewire in the renal pelvis	4	No need of intervention (1) Required more than 2 dilatations (1) Open pyeloplasty (1) Loss of renal function and involution (1)
	Persistence of hourglass image	24	HBPD and double-J stent with Prolene (21) Second dilatation with Cutting Balloon (3)

In the early postoperative period, 23 patients (22.8%) visited the emergency room to rule out a urinary tract infection, requiring an early double-J stent removal in only two cases [Clavien-Dindo III-b (22)]. Five patients required readmission because of pain and vomiting, and two due to persistent hematuria (Clavien-Dindo I).

On those patients with a successful first intervention (101 patients), the double-J stent and UPJ calibration was performed after 39.1 ± 13.7 days. Mean operative time was 18.4 ± 13.8 min, with no intraoperative complications. In 24 patients a residual stenosis was found, and in four it was not possible to access the pelvis with the guidewire. Outcome of these patients is shown in Table 3. The double-J stent with a Prolene suture, placed on those with a weak inflammatory stenosis (*n* = 21), was removed 1 week later in the outpatient clinic, without incidences.

Follow-up ultrasound results are shown in Tables 4, 5. There was a statistically significant improvement in the reduction of postoperative APD, parenchymal thickness and PCR on those patients who required one or two ERBD (*p* < 0.05, *t*-test). Furthermore, there were no statistically significant differences on those variables between patients who required one or two dilatations (*p* > 0.05). Nine patients presented a late recurrence of UPJO despite the initial success, and an additional intervention was needed (mean of 12.4 postoperative months): an additional HPB dilatation (*n* = 3), a percutaneous endopyelotomy (*n* = 3), open pyeloplasty (*n* = 2) and nephrectomy due to loss of renal function (*n* = 1). Mean ultrasound measures of these nine patients at six postoperative months were: APD = 37.0 ± 9.9 mm, PCR = 5.9 ± 2.3 and PI = −8.5 ± 61.4%.

**Table 4:** Follow-up ultrasounds: antero-posterior renal pelvis diameter.

**Final outcome**	***N* (%)**	**Pre-OP APD renal pelvis**	**Post-OP APD renal pelvis**			***p*-value**	**Percentage of improvement in APD (%)**		
			**3 m**	**6 m**	**1 y**	**1 y**	**3 m**	**6 m**	**1 y**
Resolution after one dilatation	86 (76.8%)	24.0 ± 8.9	15.8 ± 8.3	12.7 ± 6.6	9.9 ± 5.3	<0.001	30.7 ± 38.4	46.7 ± 27.1	54.9 ± 24.9
Resolution after two dilatations	11 (9.8%)	31.0 ± 13.4	21.2 ± 10.2	19.9 ± 8.5	12.1 ± 5.5	<0.001	25.4 ± 33.2	32.8 ± 25.5	56.9 ± 20.8
Required more than two dilatations, percutaneous endopyelotomy or open pyeloplasty	13 (11.6%)	28.8 ± 10.6	32.8 ± 13.8	29.1 ± 16.5	23.2 ± 16.8	0.30	−5.3 ± 46.1	0.45 ± 65.7	16.8 ± 62.7

*Patients that finally underwent a nephrectomy or kidney involution (n = 2) not included. P-value shows the significance between the preoperative ultrasound and after 1 year of follow-up. Pre-OP, pre-operative; Post-OP, post-operative*.

**Table 5:** Follow-up ultrasounds: parenchymal thickness and pelvis/cortex ratio.

**Final outcome**	***N* (%)**	**Pre-OP parenchymal thickness**	**Pre-OP PCR**	**Post-OP parenchymal thickness**			***p*-value**	**Post-OP PCR**			***p*-value**
				**3 m**	**6 m**	**1 y**	**1 y**	**3 m**	**6 m**	**1 y**	**1 y**
Resolution after one dilatation	86 (76.8%)	4.4 ± 1.7	6.7 ± 4.4	6.5 ± 2.4	7.1 ± 2.3	10.1 ± 4.8	<0.001	3.0 ± 2.2	2.1 ± 1.3	1.3 ± 0.98	<0.001
Resolution after two dilatations	11 (9.8%)	3.6 ± 0.82	9.4 ± 5.1	5.3 ± 1.6	6.7 ± 1.2	8.5 ± 4.5	0.007	4.4 ± 2.4	3.0 ± 1.1	1.8 ± 1.2	<0.001
Required more than two dilatations, percutaneous endopyelotomy or open pyeloplasty	13 (11.6%)	4.2 ± 1.4	7.6 ± 4.3	5.8 ± 1.9	5.8 ± 1.2	8.5 ± 4.2	0.011	6.4 ± 4.0	5.2 ± 3.4	3.6 ± 3.6	0.036

*Patients that finally underwent a nephrectomy or kidney involution (n = 2) not included. p value shows the significance between the preoperative ultrasound and after 1 year of follow-up. Pre-OP, pre-operative; Post-OP, post-operative*.

**Figure 2:**
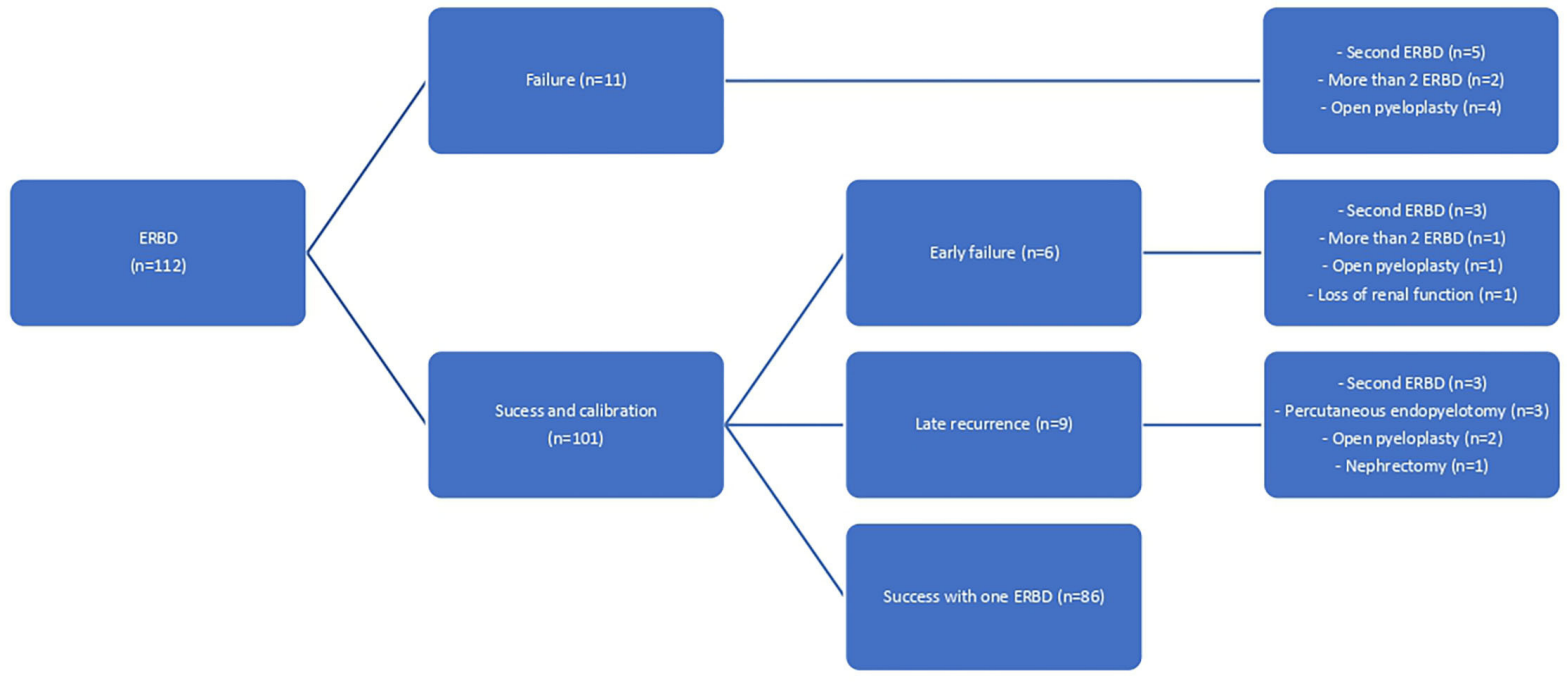
Long and short-term outcomes.

Outcomes of patients included in the study are shown in Figure 2. From the initial 112 patients, 76.8% of them (*n* = 86) required only one ERBD for the UPJO resolution. This percentage raises up to 86.6% (97 patients) if those who required a second ERBD are included. Only seven patients (6.3%) finally underwent an open pyeloplasty (median of three postoperative months, range 1–7), and two patients (1.8%) presented a total loss of renal function (one presented a pre-operative renal function of 21%, and the other, a severe pre and postoperative pyelonephritis). The other six patients were treated with more than 2 ERBD (*n* = 3) or with a percutaneous endopyelotomy (*n* = 3).

From the 103 patients who did not require an open pyeloplasty, 40 presented almost one sonographic criteria for poor prognosis, requiring 15 of them (37.5%) an additional intervention due to an obstructive diuretic drainage curve in the MAG-III renogram. From the 63 patients without any of these findings, only 2 (3.2%) required an additional ERBD.

## Discussion

In this study, we present our experience with the endourologic treatment of the congenital primary UPJO. The HPB dilatation is the core of the treatment, but the inclusion of the CB allowed a successful outcome in several cases with persistent stenosis despite the HPB inflation. We have previously reported good outcomes with the HPB (5) and the CB dilatation (18). In the present series, we report a success rate of 76.8% with one ERBD, and 86.6% when two ERBD where needed, which demonstrates the consistency of this technique in a significant number of patients and with a wide follow-up.

The minimally invasive options for the treatment of the UPJO have become more attractive in the recent years. Laparoscopic (23) and robot-assisted pyeloplasty had become more popular, showing promising results (3, 24). But concerns about its application in small children, a higher cost, the need of specific technology and demanding training make these options not universally available and applicable. In comparison, ERBD presents some advantages, as the reduction in the operative time and hospital stay [mean operative time of 240 min and 1.5–3.0 days of hospital stay in recent laparoscopic and robotic series (3, 7, 23)], less analgesics requirements (including opioids), the aesthetic benefits of the absence of scars, and its safe application in small infants. The complication rate is comparable, being the urinary infection the most frequent event. This could be related to the double-J placement, but we believe that its placement is imperative in order to avoid an acute postoperative UPJ obstruction (17). Finally, even that our success rate is slightly lower than the laparoscopic/robotic techniques, we strongly believe that the advantages of the endourologic approach make it an attractive and safe option in the treatment of the UPJO. However, as this technique does not alter the external anatomy of the ureter or renal pelvis, the surgical field is intact in case of needing a pyeloplasty.

Previous publications showed inconsistent results of the ERBD in children. Sugita et al. (25) reported a 47% success rate, and Wilkinson et al. (6) a successful treatment (combining retrograde and anterograde access) in 13/14 patients. Some authors, as Veenboer et al. (26), prefer the percutaneous anterograde approach, with reports of a high success in UPJO recurrences after pyeloplasty. In our experience, the improvement and adaptation of the endourologic tools to the pediatric population (especially with the use of <4 Fr profile instruments), made the retrograde approach a safe, less invasive and feasible option even in infants, as we demonstrate with the low percentage of intraoperative complications reported. Due to the higher risk of complications, we prefer to reserve this therapeutic option for patients with UPJO recurrence (16).

Probably the main disadvantage of this procedure is that, in some cases (23% of our patients), a second dilatation procedure under general anesthesia is needed to achieve a persistent resolution of the stenosis. In our series, no anesthetic adverse event was recorded, mainly due to the shortness and minimal invasiveness of the procedures (with a mean surgical time <30 min). Moreover, the learning curve is as needed as any other minimally invasive technique. Most part of our failures and recurrences were registered in the first half of the study, with a significative reduction of the number of failures in the last years (11 cases of failure or recurrences before year 2011, vs. 4 after that year).

Radiation exposure of the infants is an important concern in our practice. During the intervention, the operator reduces the effective dose to the minimum, and it is promoted the use of radiation shields where possible. Following the recommendations of recent data (27–30), we have changed the postoperative image follow-up protocol and the postoperative MAG-III diuretic renogram is only performed on those patients with postoperative ultrasound worsening. Following this protocol, in up to 61 patients the MAG-3 diuretic renogram could have been avoided.

This study has the limitations of a retrospective design, without a control group. The learning curve of the technique is included in the long period of time for data collection, which could affect the outcome of the first patients included. It represents a single center experience, so prospective comparative studies should be encouraged to provide definitive evidence of this procedure. However, its success lies in the use of adequate endoscopic material suitable for pediatric age, the selection of appropriate hydrophilic guidewires (0.014”–0.018”), balloon catheters with low profile and proper double-J stents for small children.

## Conclusion

The ERBD represents the least invasive option for the treatment of the congenital primary ureteropelvic junction obstruction. The outcome is acceptable with a very low rate of complications.

## Data Availability Statement

The raw data supporting the conclusions of this article will be made available by the authors, without undue reservation.

## Ethics Statement

The studies involving human participants were reviewed and approved by Normas de manejo de pacientes pediátricos, Comité Deontológico Hospital Gregorio Marañón. Written informed consent to participate in this study was provided by the participants' legal guardian/next of kin.

## Author Contributions

All authors contributed to the design of the research, data collection, analysis of the results, and writing of the manuscript. All authors contributed to the article and approved the submitted version.

## Conflict of Interest

The authors declare that the research was conducted in the absence of any commercial or financial relationships that could be construed as a potential conflict of interest.

## Publisher's Note

All claims expressed in this article are solely those of the authors and do not necessarily represent those of their affiliated organizations, or those of the publisher, the editors and the reviewers. Any product that may be evaluated in this article, or claim that may be made by its manufacturer, is not guaranteed or endorsed by the publisher.

## Abbreviations

APD: Antero-posterior renal pelvis diameter
CB: Cutting Balloon™
ERBD: Endoscopic Retrograde Balloon Dilatation
HPB: High Pressure Balloon
PCR: pelvis/cortex ratio
PI: percentage of improvement
UPJ: Ureteropelvic Junction
UPJO: Ureteropelvic Junction Obstruction.
